# Machine Learning-Based Spectral Analyses for *Camellia japonica* Cultivar Identification

**DOI:** 10.3390/molecules30030546

**Published:** 2025-01-25

**Authors:** Pedro Miguel Rodrigues, Clara Sousa

**Affiliations:** CBQF—Centro de Biotecnologia e Química Fina—Laboratório Associado, Escola Superior de Biotecnologia, Universidade Católica Portuguesa, Rua de Diogo Botelho 1327, 4169-005 Porto, Portugal; pmrodrigues@ucp.pt

**Keywords:** chemometrics, feature selection, machine learning, infrared spectroscopy, plant typing

## Abstract

*Camellia japonica* is a plant species with high cultural and biological relevance. Besides being used as an ornamental plant species, *C. japonica* has relevant biological properties. Due to hybridization, thousands of cultivars are known, and their accurate identification is mandatory. Infrared spectroscopy is currently recognized as an accurate and rapid technique for species and/or subspecies identifications, including in plants. However, selecting proper analysis tools (spectra pre-processing, feature selection, and chemometric models) highly impacts the accuracy of such identifications. This study tests the impact of two distinct machine learning-based approaches for discriminating *C. japonica* cultivars using near-infrared (NIR) and Fourier transform infrared (FTIR) spectroscopies. Leaves infrared spectra (NIR—obtained in a previous study; FTIR—obtained herein) of 15 different *C. japonica* cultivars (38 plants) were modeled and analyzed via different machine learning-based approaches (Approach 1 and Approach 2), each combining a feature selection method plus a classifier application. Regarding Approach 1, NIR spectroscopy emerged as the most effective technique for predicting *C. japonica* cultivars, achieving 81.3% correct cultivar assignments. However, Approach 2 obtained the best results with FTIR spectroscopy data, achieving a perfect 100.0% accuracy in cultivar assignments. When comparing both approaches, Approach 2 also improved the results for NIR data, increasing the correct cultivar predictions by nearly 13%. The results obtained in this study highlight the importance of chemometric tools in analyzing infrared data. The choice of a specific data analysis approach significantly affects the accuracy of the technique. Moreover, the same approach can have varying impacts on different techniques. Therefore, it is not feasible to establish a universal data analysis approach, even for very similar datasets from comparable analytical techniques.

## 1. Introduction

*Camellia japonica* is an evergreen shrub renowned for its vibrant blooms, holding significant cultural relevance. In many Asian cultures, particularly Japan and China, the camellia flower symbolizes longevity, prosperity, and good fortune. Its association with beauty and refinement has made it a popular choice for gardens, art, and literature throughout history. Beyond its aesthetic appeal, *C. japonica* is also recognized for its biological properties and economic relevance [1]. Its oil, extracted from the seeds, is used in various cosmetic and skincare products due to its moisturizing and antioxidant properties. Also, the plant’s wood is highly appreciated for its durability and is used in woodworking and carpentry. Thousands of *C. japonica* cultivars are known, and this number is constantly increasing due to the development of new cultivars through hybridization and selection [2]. Cultivar discrimination is crucial in preserving genetic diversity, accurate plant labeling, and selection; it helps plant breeders develop and improve cultivars and contributes to maintaining the cultural heritage, such as some cultivars’ cultural and historical significance. However, accurate discrimination is challenging due to their similar appearances, and a combination of characteristics and/or methods is usually employed. The most evaluated characteristics are flowers and leaf colors, form, and size, as well as bloom time and growth habitat, sometimes combined with DNA analysis techniques [3,4]. The operator’s decision limits the visual inspection of plant characteristics, while DNA analysis is a relatively expensive and laborious technique. Alternative methods, such as those based on vibrational spectroscopy, encompassing near-infrared (NIR) and Fourier transform infrared (FTIR) spectroscopies, are already recognized as accurate techniques for species and infra-species discrimination of plants [1,5,6] and bacteria [7,8]. Nevertheless, data analysis plays a significant role in the process of discrimination and/or identification, which highly impacts the success rates of the spectroscopic technique. The development of sophisticated computers and data analysis methods in the last two to three decades has led to many multivariate data analysis tools, including some freely available online ones. The choice of the best data analysis tools needs to be judicious and adapted to the type of data and goal of work. Machine learning methods are among the most exploited tools used to analyze spectroscopic data, enabling computers to learn from data and improve their performance on a specific task without being explicitly programmed. These methods encompass various algorithms and techniques, typically requiring human intervention to extract relevant features from data. It uses relatively simple models, such as linear regression, decision trees, or support vector machines, and can be effective with relatively small datasets of structured data. Applying these methods to spectroscopic data usually precedes a feature selection method and/or spectral pre-processing to identify the most relevant features from the dataset to improve model performance and reduce computational cost. It can be implemented by applying specific data statistical analysis algorithms for feature selection (i.e., analysis of variance F-value (ANOVA), chi-squared test, false discovery rate (FDR), mutual information, and family-wise error rate (FER)), or by simply dividing the spectra wavelength range into well-defined intervals.

Later, deep learning-based approaches were developed to overcome the limitations associated with spectral pre-processing methods, and their use became quite popular. However, there are still some limitations to consider. For instance, deep learning models often require large amounts of labeled data and significant computational resources, which can be a barrier for some applications. Additionally, these models can be seen as “black boxes”, making it difficult to interpret the results and understand the underlying decision-making process. That is why machine learning feeds with data obtained from spectral analyses still are used routinely for classification proposes [9].

In this context, this work aims to

1. Test two distinct machine learning-based approaches for discriminating *C. japonica* cultivars using NIR and FTIR spectroscopy, respectively, as follows:
  - Approach 1: Select spectral ranges based on known absorption bands of biological molecules and apply a partial least squares discriminant analysis (PLSDA) for classification.
  - Approach 2: Use forward feature selection taking advantage of five statistical selectors (ANOVA, chi-squared, FDR, mutual information, and FER), followed by principal component analysis (PCA) and testing multiple classifiers for prediction.
2. Compare and discuss the performance of the two infrared-based techniques together with both data analyses applied approaches by analyzing their capability to accurately identify each one of the studied *Camellia japonica* cultivars.

## 2. Materials and Methods

### 2.1. Camellia japonica Samples

*C. japonica* leaves of 38 different plants belonging to 15 cultivars (Table 1) were collected at Viveiro da Câmara Municipal do Porto (VMP), GPS: 41.155830, −8.558920 and at Jardim Botânico do Porto (JBP), GPS: 41.153650, −8.642528. Ten leaves per plant were collected twice within one month for 20 leaves per plant. Immediately after collection, leaves were transported to the laboratory, rinsed with distilled water, and dried with tissue paper. Leaves were allowed to air-dry at room temperature, avoiding daylight exposure, until no difference in mass was observed. The air-dried leaves collected for each plant (20 leaves) were milled through a coffee mill (MS 50, Taurus, Oliana, Spain) and sieved. The fine powder obtained for each plant was transferred to borosilicate flasks until spectral acquisition (no more than 2 days). See the Supplementary Materials for additional information regarding *C. japonica* cultivars identification (including photos, first author names and plant descriptions). Detailed maps of and JBP were also included, as well as specific plant location in the corresponding gardens.

### 2.2. Infrared Spectra Acquisition

**Figure 1:**
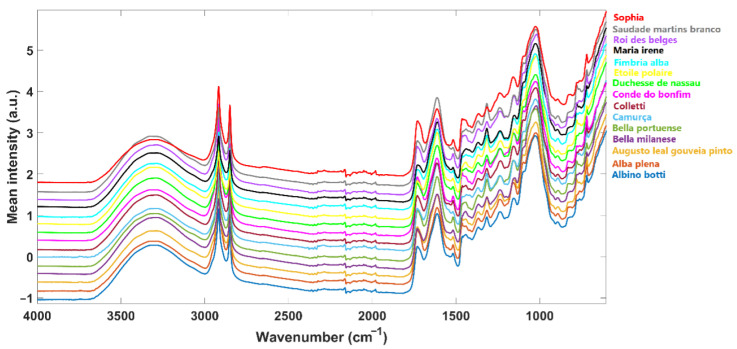
*C. japonica* air-dried leaves FTIR-ATR spectra (mean spectra of each cultivar).

Fourier transform infrared with attenuated total reflectance (FTIR-ATR) spectra of *C. japonica* air-dried leaves were acquired on a Fourier transform PerkinElmer Spectrum BX FTIR Systemspectrophotometer (Waltham, MA, USA) with a DTGS detector. Spectra were acquired in diffuse reflectance mode through a PIKE Technologies Gladi ATR accessory (Madison, WI, USA) from 4000 to 600 cm_−1_, with a 4 cm_−1_ resolution. Each spectrum resulted from 32 scan co-additions. A small portion of the powder was transferred to the ATR crystal for each sample, and a pressure of 150 N·cm_−2_ was applied. This procedure was repeated three times for each sample. The ATR crystal was cleaned, and a background was acquired between each sample. NIR spectra were previously acquired. For details, please see the study of Sousa et al. 2024 [5]. Both spectra were stored in an Excel file of 228 entries (spectral data of 15 *C. japonica* cultivars) with a spectral resolution of 1556 bins for NIR data and 1701 for FTIR-ATR data. The mean FTIR-ATR spectra of each class are presented in Figure 1.

### 2.3. Data Analysis and Prediction

**Figure 2:**
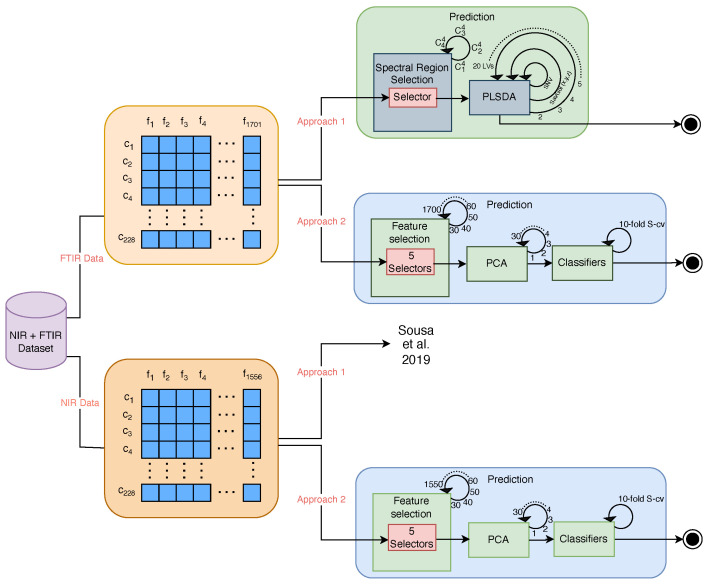
Data analysis and prediction workflow. The Approach 1 workflow for NIR Data (Sousa et al. 2019) can be found at [5].

Two different approaches (Approach 1 and Approach 2) were used for *C. japonica* cultivar prediction. Approach 1 is a more classical method that involves selecting spectral regions in a combined way without blinding to feed PLSDA models. The second approach involves blinding the selection of the spectral frequency using statistical methods for forward feature selection to feed a set of seven machine learning models. An overview of the methods used is shown in Figure 2.

#### 2.3.1. Approach 1—Unblinding Spectral Region Selection Combined with PLSDA

FTIR-ATR spectra were analyzed using partial least squares discriminant analysis (PLSDA) [10,11]. PLSDA was used as the supervised model to develop discrimination models. Each *C. japonica* cultivar was assigned to a different class. Data were divided into two datasets (70% for calibration and 30% for validation) in a random mode. Still, unbalanced classes were avoided in the calibration and validation sets, and all *C. japonica* cultivars were guaranteed in both sets. Therefore, two spectra were calibrated from the three spectra obtained for each sample, and one spectrum was validated. The leave-one-sample-out procedure was used to estimate the optimum number of latent variables (LVs) using only the calibration set (tested 2–20 LVs). The optimal number of LVs was selected based on a compromise between the highest percentage of correct predictions and the lowest number of LVs (when the variation in the accuracy rate between two consecutive LVs was less than 5%, the lowest number of LVs was selected). FTIR spectra were divided into four regions to identify the best spectral region: R1 from 3000 to 2800 cm_−1_, R2 from 1800 to 1500 cm_−1_, R3 from 1500 to 1200 cm_−1_, and R4 from 1200 to 900 cm_−1_. All these regions (four) were tested individually and in all possible combinations, generating 15 possible combinations. Several pre-processing techniques, namely the standard normal variate (SNV) [12] and a Savitzky–Golay (SG) filter [13] (x, y, z; where x is the filter width, y is the polynomial order and z is the derivative used), were also tested, individually and in all possible combinations to find the best pre-processing technique. After finding the best spectral region and pre-processing technique, the PLSDA calibration model projected the validation set to assess the percentage of correct predictions for each *C. japonica* cultivar. The predictions of PLSDA models were expressed in the form of confusion matrices, where the sum of the diagonal elements gives the total percentage of correct predictions [14]. Matlab version R2023a (MathWorks, Natick, MA, USA) and the PLS Toolbox version 9.2.1 (Eigenvector Research Incorporated, Manson, WA, USA) were used for all the calculations.

NIR spectra were analyzed using the same workflow. For details, please see Sousa et al.’s study of 2019 [5].

#### 2.3.2. Approach 2—Blinding Forward Spectral Frequencies’ Selection Combined with Scikit-Learn ML Models

Infrared data were loaded into Python (version 3.9.12, Python Software Foundation, Wilmington, DE, USA) and normalized using the min–max method to scale the NIR and FTIR-ATR spectra. To enhance the discrimination power for identifying *Camellia japonica* classes, an iterative process was employed to select frequency bins within the spectra. Five feature selectors—ANOVA, chi-squared, FDR, mutual information, and FER—were used to choose frequency bins from 70 to 1550 for NIR and from 70 to 1710 for FTIR-ATR, in steps of 10. Principal component analysis (PCA) was then applied to these selected bins to retrieve the most important components, ranging from 1 to 30. This approach ensures that the most relevant features are identified and analyzed, maximizing the discrimination power for classifying *Camellia japonica*. By combining multiple feature selection methods and PCA, the process effectively reduces dimensionality while retaining significant variance, leading to more accurate and robust classification results.

**Figure 3:**
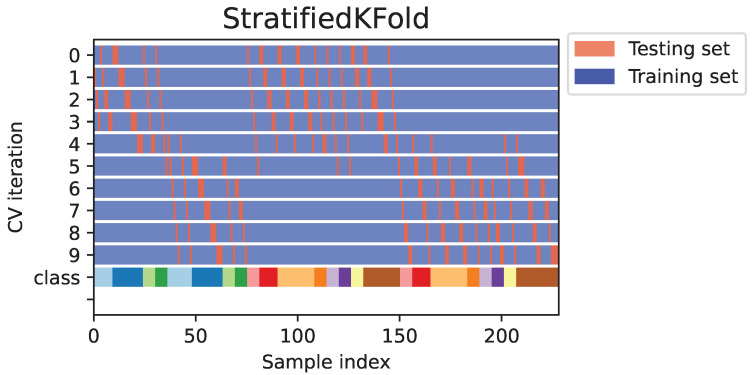
The stratified k-fold strategy used for classifying data by ML models (Approach 2).

During the iterative process of FF-PCA, data were presented to the entries of 10 predesigned scikit-learn ML models [15] with different configurations (see Table 2 for more information). A stratified 10-fold cross-validation process was employed, as illustrated in Figure 3, to identify the best model for the discrimination task. This method ensures that each fold of the dataset maintains the same proportion of class labels, providing a more reliable estimate of model performance compared to standard k-fold cross-validation [16]. The Python code for Approach 2 was upload to GitHub and is freely available at https://github.com/pmrodri/Cammelia-Dataset.git, accessed on 22 January 2025 (including the dataset).

## 3. Results

**Figure 4:**
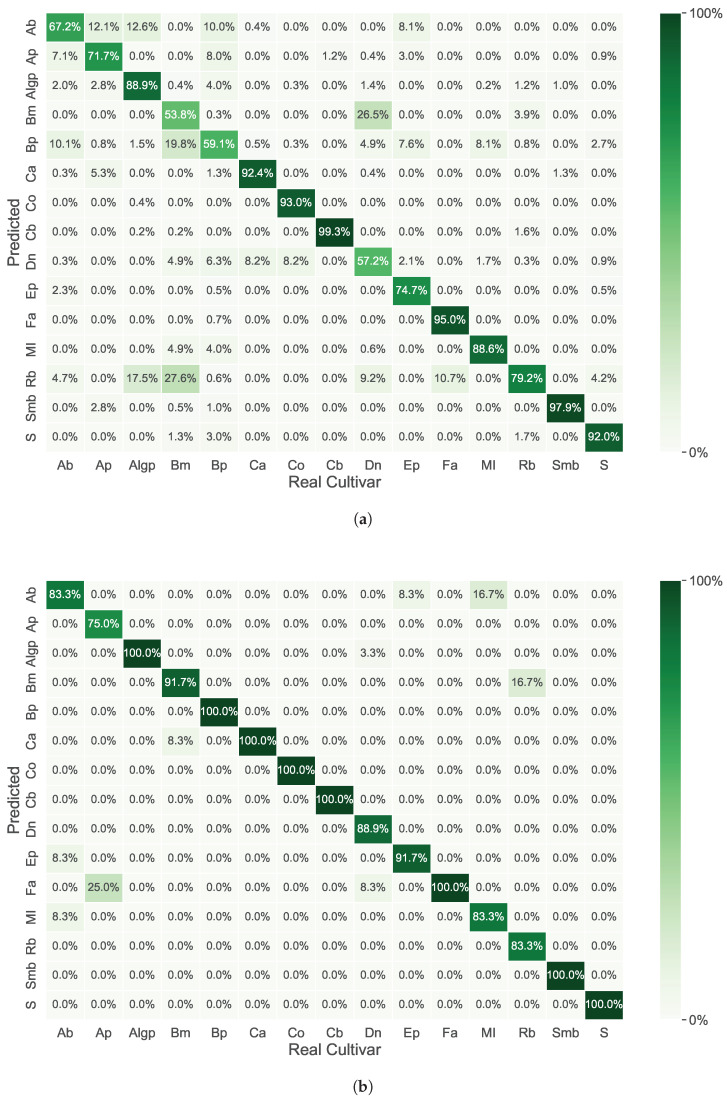

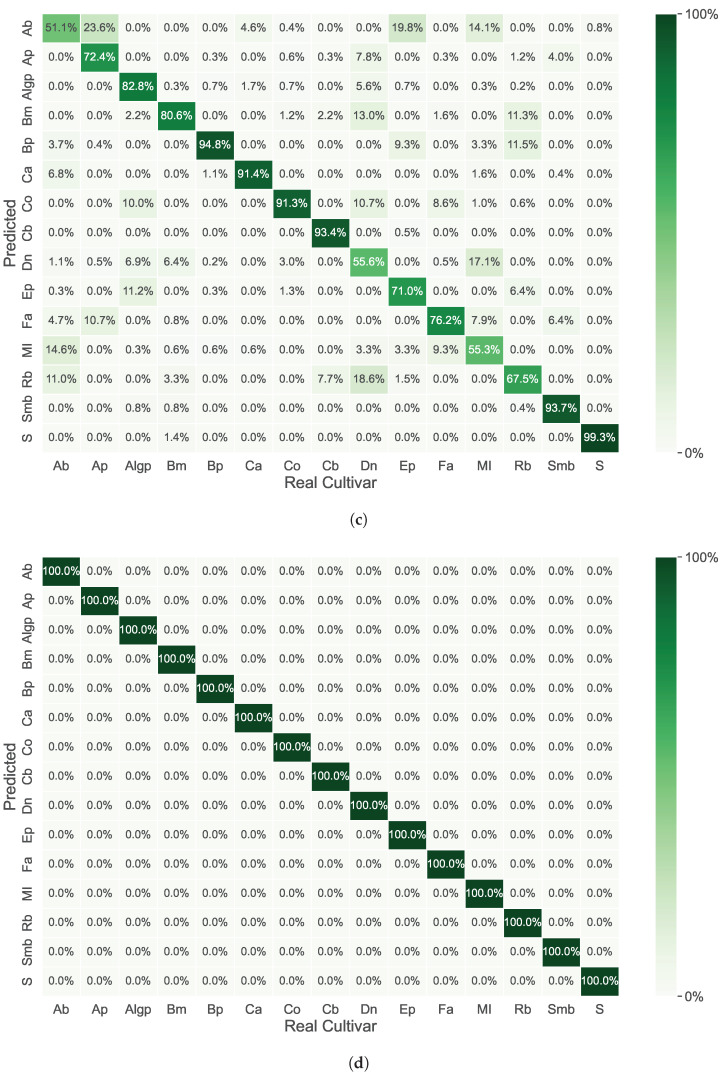
Confusion matrices with prediction accuracy in percentage (%) of the discrimination processes per each approach and used data modality (NIR and FTIR-ATR). (**a**) Discrimination results obtained in Sousa et al. 2019 study date from [5]; (**b**) NIR—Approach 2: GaussianNB discrimination model (10-fold SCV); 13 PCA components of 710 frequency bins selected by FDR forward selection. (**c**) FTIR-ATR—Approach 1: PLSDA discrimination model [17 LVs; 1800–900 cm_−1_; pre-processing: SNV+SavGol(15,2,2)]. (**d**) FTIR—Approach 2: BaggingClassifier discrimination model (10-fold SCV); 7 PCA components of 640 frequency bins selected by FER forward selection. Ab—Albino botti; Ap—Alba plena; Algp—Augusto leal gouveia pinto; Bm—Bella milanese; Bp—Bella portuense; Ca—Camurça; Co—Colletti; Cb—Conde do bonfim; Dn—Duchesse de nassau; Ep—Etoile polaire; Fa—Fimbria alba; MI—Maria irene; Rb—Roi des belges; Smb—Saudade martins branco; S—Sophia.

In Figure 4, the discrimination results of *C. japonica* cultivars per each approach and used data modality (NIR and FTIR) are illustrated as confusion matrices.

Figure 4a presents the correct cultivar assignments obtained with the NIR spectra presented in the study of Sousa et al. 2019 [5] (just for a comparison with the results presented). The authors used an 83.4% [spectral region between 6076 and 5380 cm_−1_ and 4956–4030 cm_−1_ with 17LVs and a pre-processing combination of SNV and SavGol(17,2,1)] to achieve the best discrimination results. The best predicted cultivar was Conde do bonfim (99. 0%), while the worst one was Bella milanese (52.4%).

Figure 4b shows the correct cultivar assignments obtained in the present study using NIR spectra data (Approach 2). These results were achieved using the best iterative pair of FFs-PCA, with a GaussianNB discrimination model fed with the top 13 PCA components derived from 710 NIR frequency bins, selected from a total of 1556 through FDR forward selection within a 10-fold SCV. The FDR selector was shown to be the best feature selector for this problem. The trained GaussianNB model (1) perfectly identified 8 species (Augusto leal gouveia pinto, Bella portuense, Carmurça, Colletti, Conde bonfim, Saudade martins branco, Fimbria alba, Sophia) out of 15; (2) achieved discrimination accuracies higher than 80% for 14 out of 15 species; and (3) was less accurate for Alba plena, with a prediction accuracy of 75%.

Figure 4c presents the optimum PLSDA model’s results for *C. japonica* cultivar discrimination with FTIR-ATR spectra—Approach 1—obtained with a spectral region between 1800 and 900 cm_−1_ with 17 LVs and a pre-processing combination of SNV and SavGol (15,2,2). The total percentage of correct cultivar assignments was 79.0%. The best-predicted cultivars (higher than 90% of accurate predictions) were Bella portuense, Camurça; Colletti, Conde do bonfim; Saudade martins branco; and Sophia (the best predicted one achieved 99.3%). The worst predicted cultivars (lower than 60% of correct assignments) were Maria Irene, Duchesse de Nassau, and Albino botti (the worst predicted one obtained 51.1%).

Figure 4d illustrates the accurate cultivar assignments achieved in this study using FTIR-ATR spectral data (Approach 2). These results were obtained by employing the optimal iterative combination of FFs-PCA with a GaussianNB discrimination model. This model was trained using the top 13 PCA components derived from 640 FTIR-ATR frequency bins. These bins were selected from a total of 1701 through FER forward selection within a 10-fold SCV. FER forward selection showed to be the best selector. The trained BaggingClassifier model successfully identified all the species involved.

**Figure 5:**
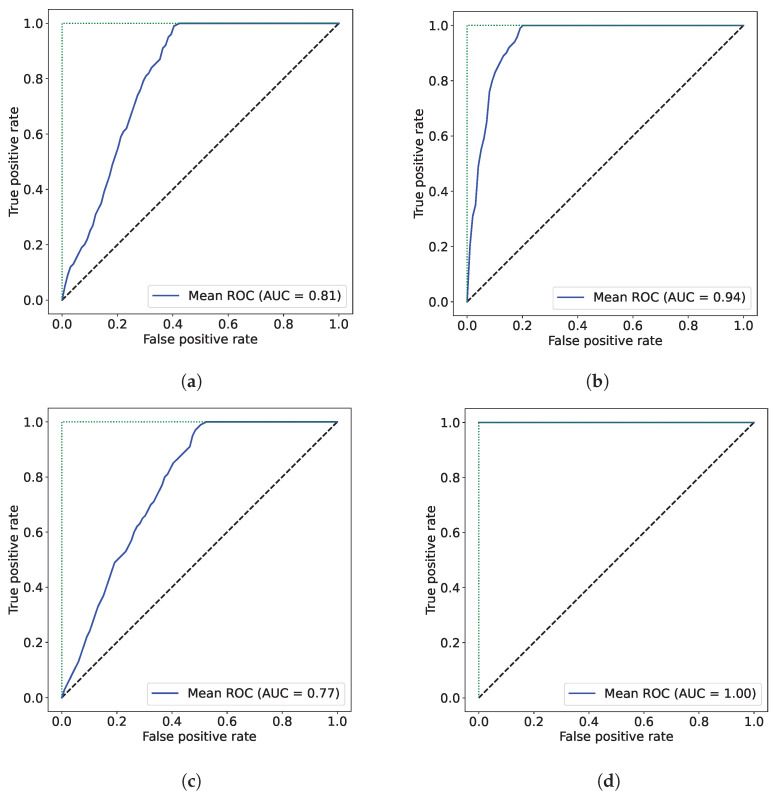
ROC curves of various applied approaches, highlighting the mean AUC. (**a**) ROC curve—discrimination results obtained in Sousa et al. 2019 study [5]. (**b**) ROC curve NIR—Approach 2. (**c**) ROC curve FTIR-ATR—Approach 1. (**d**) ROC curve FTIR-ATR—Approach 2.

The associated ROC curves for Figure 4 are presented in Figure 5.

## 4. Discussion

Infrared-based techniques for typing/classification are currently well documented and widely accepted. In the literature, a vast number of published works report the success of techniques such as near- and mid-infrared spectroscopies for bacterial [7,8], yeast [17], and plant [5,6,14,18] discrimination at different taxonomic levels. However, no studies, except one by the same authors [5], were found exploring the potential of infrared-based techniques for *C. japonica* cultivar discrimination. This lack of studies prevents a comparison for being carried out of the approaches’ accuracy developed herein for *C. japonica* cultivar discrimination. In this work, the discrimination of *C. japonica* cultivars was assessed with NIR and FTIR spectroscopies, and distinct results were obtained regarding the accuracy of the infrared technique (Table 3). Globally, the accuracy of both techniques (NIR and FTIR) was satisfactory (from 77.5% to 100.0% of correct cultivar assignments) and reasonably comparable to those obtained in similar studies [18,19,20]. The ROC curves were obtained for the four models to evaluate their performance (Figure 5). The worst model (poorer ROC curve, with AUC = 0.77), was obtained with the FTIR data and Approach 1, while the best one was achieved with the FTIR data and Approach 2 (AUC = 1). However, according to the literature, a meaningful test should have an AUC greater than 0.5, being acceptable at a value higher than 0.8, which is the case for the models developed here. However, different percentages of correct cultivar assignments were found with NIR (81.3% and 94.3%, for Approaches 1 and 2, respectively) and FTIR (77.5% and 100.0%, for Approaches 1 and 2, respectively) spectroscopies. These results corroborate other studies that have reported distinct percentages of correct assignments for different infrared-based techniques [18,21].

The results indicated that the performance varied by more than 10%, depending on the spectral modeling approach used, highlighting the impact of different machine learning methods on the outcomes. Furthermore, a higher percentage of correct *C. japonica* cultivar assignments was obtained with NIR spectroscopy when Approach 1 was used for spectral modeling. However, with Approach 2, a higher percentage of correct assignments was achieved using FTIR data. These apparently contradictory results demonstrate the relevance of the work presented herein. The selection of spectral data modeling tools highly impacts the accuracy of the utilized infrared technique. These findings are in agreement with the published literature. Li and co-workers [22] used four variable selection methods and two non-linear machine learning models to predict the wood density of *Tilia tuan* Szyszyl, *Acer mono Maxim*, *Chinese white poplar*, *Japanese elm*, and *Dahurian larsh* from different geographic regions. The authors reported a strong impact of modeling tools (variable selection + model) in the accuracy of wood density prediction for all the species studied. Moreover, different accuracies in the prediction were obtained for each species with the same modeling tools. These results emphasize the relevance of selecting modeling tools and the fact that it was not possible to find a perfect chemometric model that could be used in all scenarios. A different study conducted by Li et al. in 2023 [23] evidenced the relevance of using an appropriate variable selection method. Even using the same chemometric model, different feature selection models impact the ability of NIR to be used for predicting the total mold count (by *Aspergillus flavus*) in peanuts. Other authors [24] tested three machine learning methods (least square support vector machine, random forest, and principal component–neural network) to predict rice storage time and quality with NIR data. Huang and colleagues reported a 94.3–95.7% accuracy in the training set and an 86.7–90.0% accuracy for the test set, depending on the method used. More recently, deep learning-based approaches have been developed for system classification, allowing overpassing some limitations linked to spectral pre-processing methods. Despite not being perfect, these newly developed methods proved to be quite effective in classification. Lange et al. 2024 [25] proved that SMolNet, a classifier based on Siamese network architecture, is quite effective in comparing X-ray powder diffraction patterns even with limited training data. Wang and colleagues [26] also compared the X-ray diffraction (XDR) patterns of metal–organic frameworks through a convolutional neural network (CNN) trained with theoretical data and a quite limited experimental dataset. An additional study [27] also demonstrated that Siamese networks are well suited for data transfer between XRD datasets, achieving an accuracy of 99% even for materials not present in the training dataset. In a different context, other authors [28] developed a self-supervised learning technique that was custom-tailored for genomic data. The method proved to be effective even with nearly 10 fewer labeled training data. These distinct studies clearly demonstrate the relevance of selecting an appropriate data analysis approach.

## 5. Conclusions

This work used two vibrational spectroscopic techniques (NIR and FTIR) to discriminate *C. japonica* cultivars. Data analyses were undertaken through two different approaches (Approach 1—unblinding spectral region selection combined with PLSDA; Approach 2—blinding forward spectral frequencies selection combined with scikit-learn ML models) for spectral data modeling. Despite both being machine learning-based methods, the performance of the infrared techniques varied from 81.3% to 94.3% for NIR spectroscopy and from 77.5% to 100% for FTIR spectroscopy. Globally, Approach 2 was revealed to be the best one, with higher percentages of correct assignments for both techniques. These results emphasize the relevance of a proper data analysis selection workflow and demonstrate that no universal chemometric method works for all scenarios. The current study would benefit from incorporating additional samples for advanced deep learning-based data analysis. This will be pursued in future research. 

## Figures and Tables

**Table 1:** Details about the *C. japonica* cultivar leaves included in this study.

Cultivar	N° of Plants	Collecting Local
Albino botti	1	VMP
	1	JBP
Alba plena	1	VMP
	1	JBP
Augusto leal gouveia pinto	2	VMP
	3	JBP
Bella Milanese	2	JBP
Bella portuense	2	JBP
Camurça	2	VMP
Colletti	2	VMP
	1	JBP
Conde do bonfim	3	JBP
Duchesse de nassau	3	JBP
Etoile polaire	2	JBP
Fimbria alba	2	VMP
Maria irene	2	JBP
Roi des belges	2	JBP
Saudade martins branco	4	VMP
Sophia	2	JBP

VMP—Viveiro da Câmara Municipal do Porto; JBP—Jardim Botânico do Porto.

**Table 2:** Used scikit-learn ML classifiers and hyperparameters—Approach 2.

Classifier	Hyperparameters
AdaBoost Classifier	Default parameters (n_estimators = 50, learning_rate = 1.0, algorithm = “SAMME”)
Bagging Classifier	Default parameters (n_estimators = 10)
Decision Tree Classifier	Default parameters (Max_depth=5)
Gaussian NB	Default parameters
Quadratic Discriminant Analysis	Default parameters
K Nearest Neighbors Classifier	Default parameters (n_neighbors = 5)
Linear Discriminant Analysis	Default parameters
Logistic Regression	Default parameters (solver: “lbfgs” + max_iter = 1000)
Support-Vector Machines	Default parameters (kernel = “linear”, max_iter = 1000, and C = 1.0)
Support-Vector Machines	(kernel = “RBF”, C = 1.0, gamma = ‘scale’, probability = True)

**Table 3:** Total percentages of correct *C. japonica* cultivar assignments obtained from the optimum models with NIR and FTIR through Approach 1 and Approach 2.

	NIR	FTIR-ATR
Approach 1	81.3%	77.5%
Approach 2	94.3%	100.0%

## Data Availability

Data cannot be made available due to privacy reasons.

## Supplementary Materials

The following supporting information can be downloaded at: https://www.mdpi.com/article/10.3390/molecules30030546/s1.
