# Supplementary material for: Machine Learning-Based Spectral Analyses for Camellia japonica Cultivar Identification
Source: Molecules. 2025 Jan 25;30(3):546. doi: 10.3390/molecules30030546 (PMC11821100; doi:10.3390/molecules30030546)
Supplement: Supplementary file 1 [file molecules-30-00546-s001.zip › molecules-3343774-supplementary.pdf]

Supplementary Material

Machine Learning-Based Spectral Analysis for *Camellia japonica* Cultivar Identification

Pedro Miguel Rodrigues[1] and Clara Sousa[1,*]

CBQF---Centro de Biotecnologia e Química Fina---Laboratório Associado, Escola Superior de Biotecnologia, Universidade Católica Portuguesa, Rua de Diogo Botelho 1327, 4169-005 Porto, Portugal;

Correspondence: cssousa@ucp.pt

Table S1. Detailed information regarding *japonica* cultivars leaves identification collected at Viveiro da Câmara Municipal do Porto (VMP), GPS: 41.155830, -8.558920 and at Jardim Botânico do Porto (JBP), GPS: 41.153650, -8.642528.

Cultivar	Nº of plants	Collecting local	Garden name	Photo*,#
Albino Botti	Plant 1	VMP	Viveiro da Câmara Municipal do Porto (Figure S1)	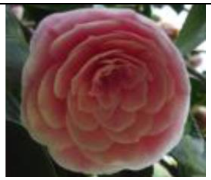
	Plant 2	JBP	Jardim do Peixe (Figure S2)	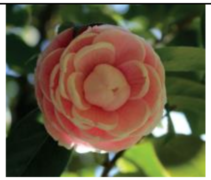
Alba Plena	Plant 1	VMP	Viveiro da Câmara Municipal do Porto (Figure S1)	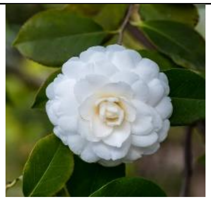
	Plant 2	JBP	Jardim do Rapaz de Bronze (Figure S3)	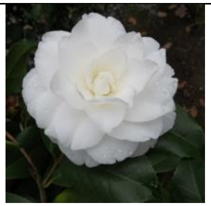

Augusto Leal Gouveia Pinto	Plant 1	VMP	Viveiro da Câmara Municipal do Porto (Figure S1)	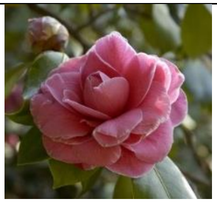
	Plant 2			
	Plant 1	JBP	Jardim do Rapaz de Bronze (Figure S3)	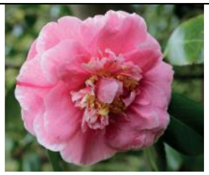
	Plant 2		Jardim do Peixe (Figure S2)	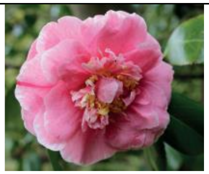
	Plant 3		Jardim das Camélias (Figure S4)	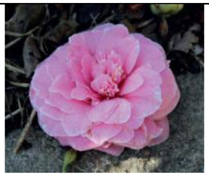
Bella Milanese	Plant 1	JBP	Jardim dos Jotas (Figure S5)	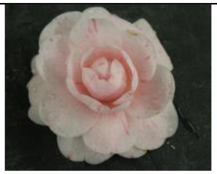

	Plant 2		Jardim das Camélias (Figure S4)	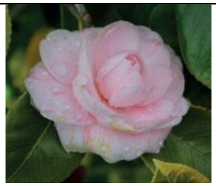
Bella Portuense	Plant 1	JBP	Jardim dos Jotas (Figure S5)	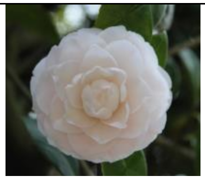
	Plant 2		Bosquete da Araucária (Figure S6)	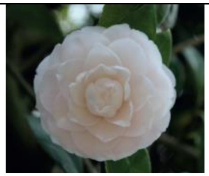
Camurça	Plant 1	VMP	Viveiro da Câmara Municipal do Porto (Figure S1)	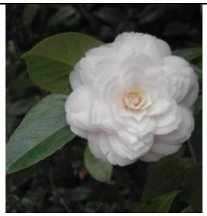
	Plant 2			
Colletti	Plant 1	VMP	Viveiro da Câmara Municipal do Porto (Figure S1)	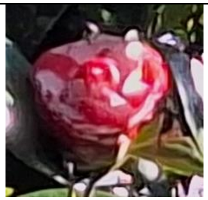
	Plant 2			

	Plant 3	JBP	Jardim dos Jotas (Figure S5)	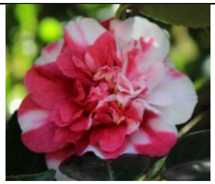
Conde do Bonfim	Plant 1	JBP	Roseiral (Figure S7)	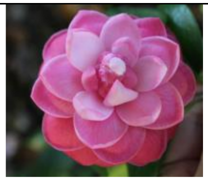
	Plant 2		Jardim dos Jotas (Figure S5)	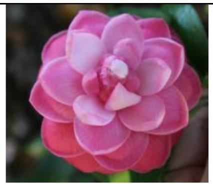
	Plant 3		Jardim do Peixe (Figure S2)	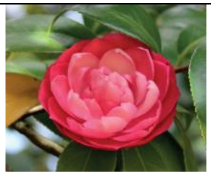
Duchesse de Nassau	Plant 1	JBP	Jardim dos Jotas (Figure S5)	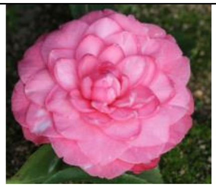

	Plant 2		Bosquete do Liquidambar (Figure S8)	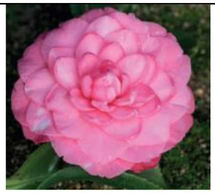
	Plant 3		Bosquete dos Cedros (Figure S9)	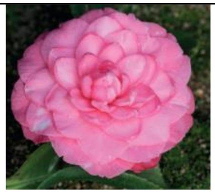
Etoile Polaire	Plant 1	JBP	Jardim do Peixe (Figure S2)	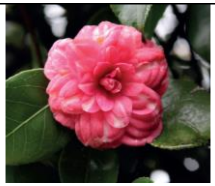
	Plant 2			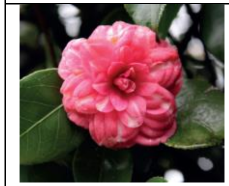
Fimbria Alba	Plant 1	VMP	Viveiro da Câmara Municipal do Porto (Figure S1)	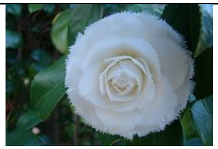
	Plant 2			

Maria Irene	Plant 1	JBP	Jardim do rapaz de bronze (Figure S3)	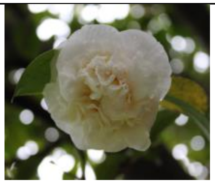
	Plant 2		Jardim do Peixe (Figure S2)	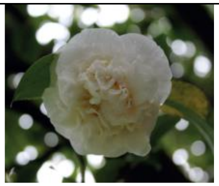
Roi des Belges	Plant 1	JBP	Jardim dos Jotas (Figure S5)	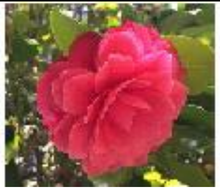
	Plant 2		Jardim do Peixe (Figure S2)	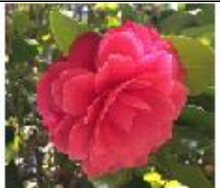
Saudade Martins Branco	Plant 1	VMP	Viveiro da Câmara Municipal do Porto (Figure S1)	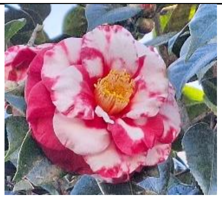
	Plant 2			
	Plant 3			
	Plant 4			

Sophia	Plant 1	JBP	Roseiral (Figure S7)	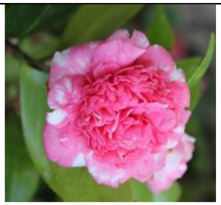
	Plant 2		Jardim dos Jotas (Figure S5)	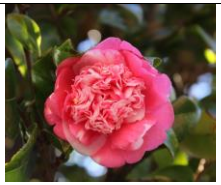

* For some cultivars, the Jardim Botânico do Porto (JBP) used the same picture for different plants (of the same cultivar) in their internal documents. In other cases, plants were individually photographed.

# Viveiro da Câmara Municipal do Porto (VMP) only provided one photo per plant cultivar.

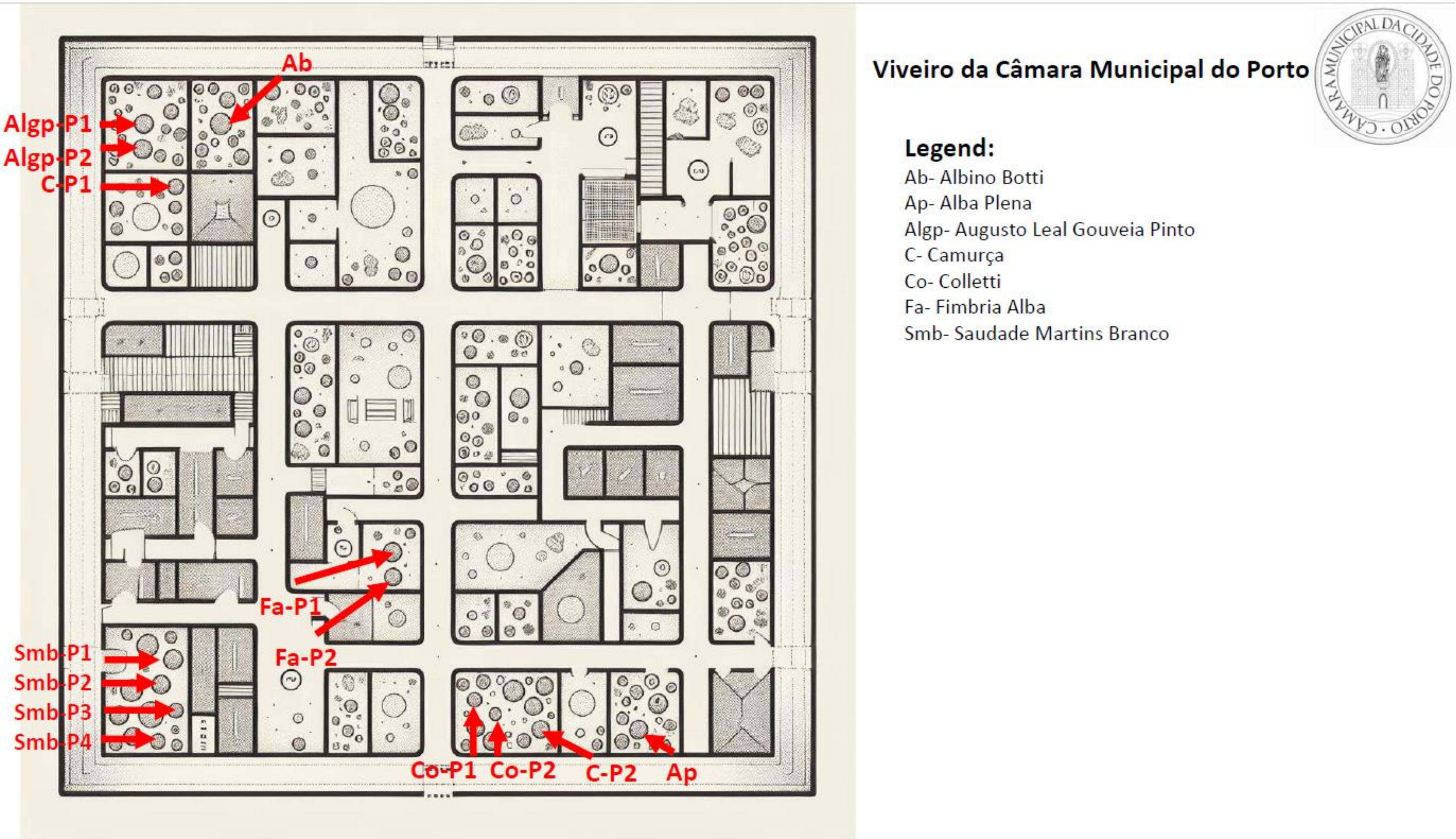

Figure S1. Detailed information about Viveiro da Câmara Municipal do Porto (VMP), GPS: 41.155830, -8.558920 and plants localization.

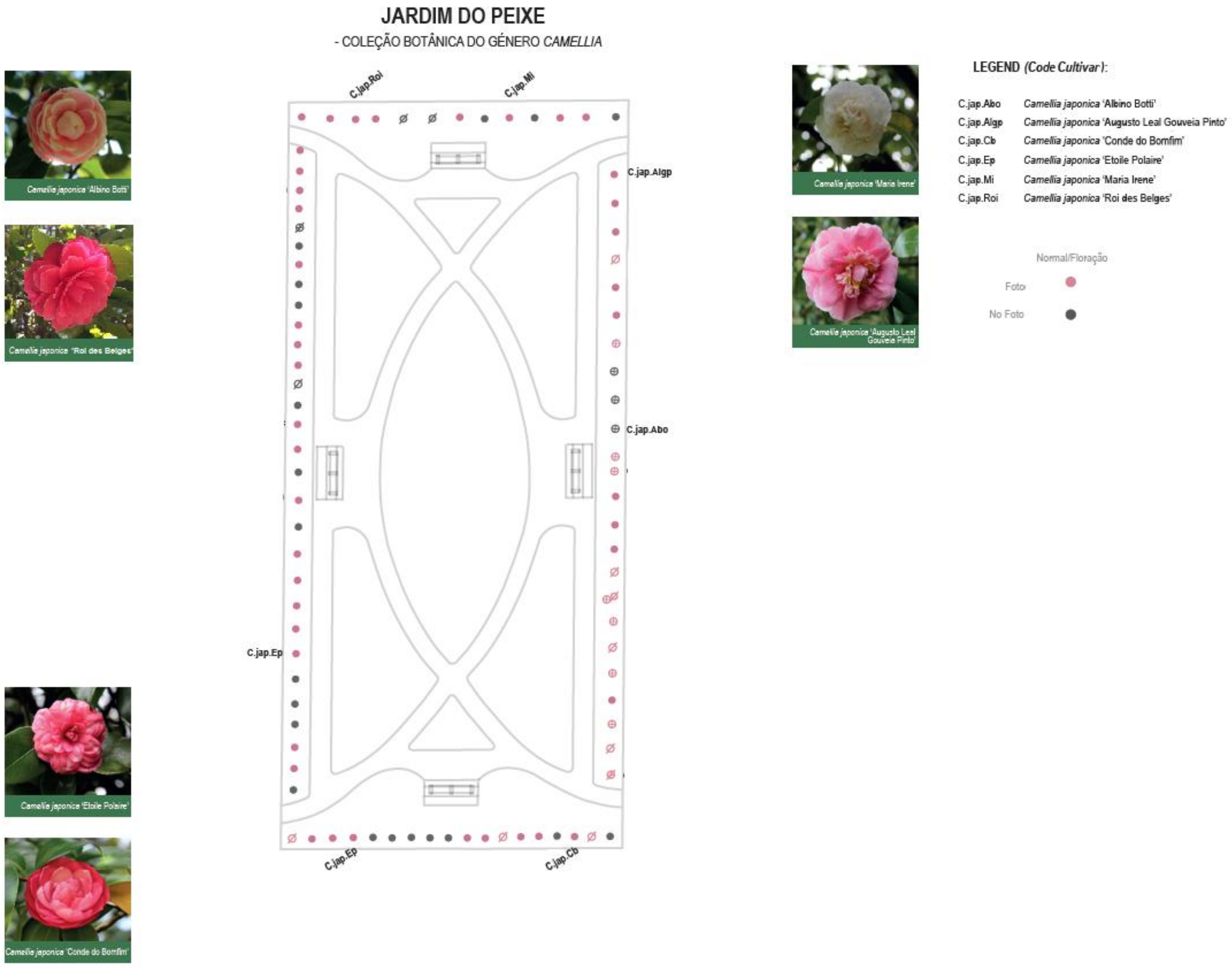

Figure S2. Detailed information about *Jardim dos Peixe* and plants localization at Viveiro da Câmara Municipal do Porto (VMP), GPS: 41.155830, -8.558920.

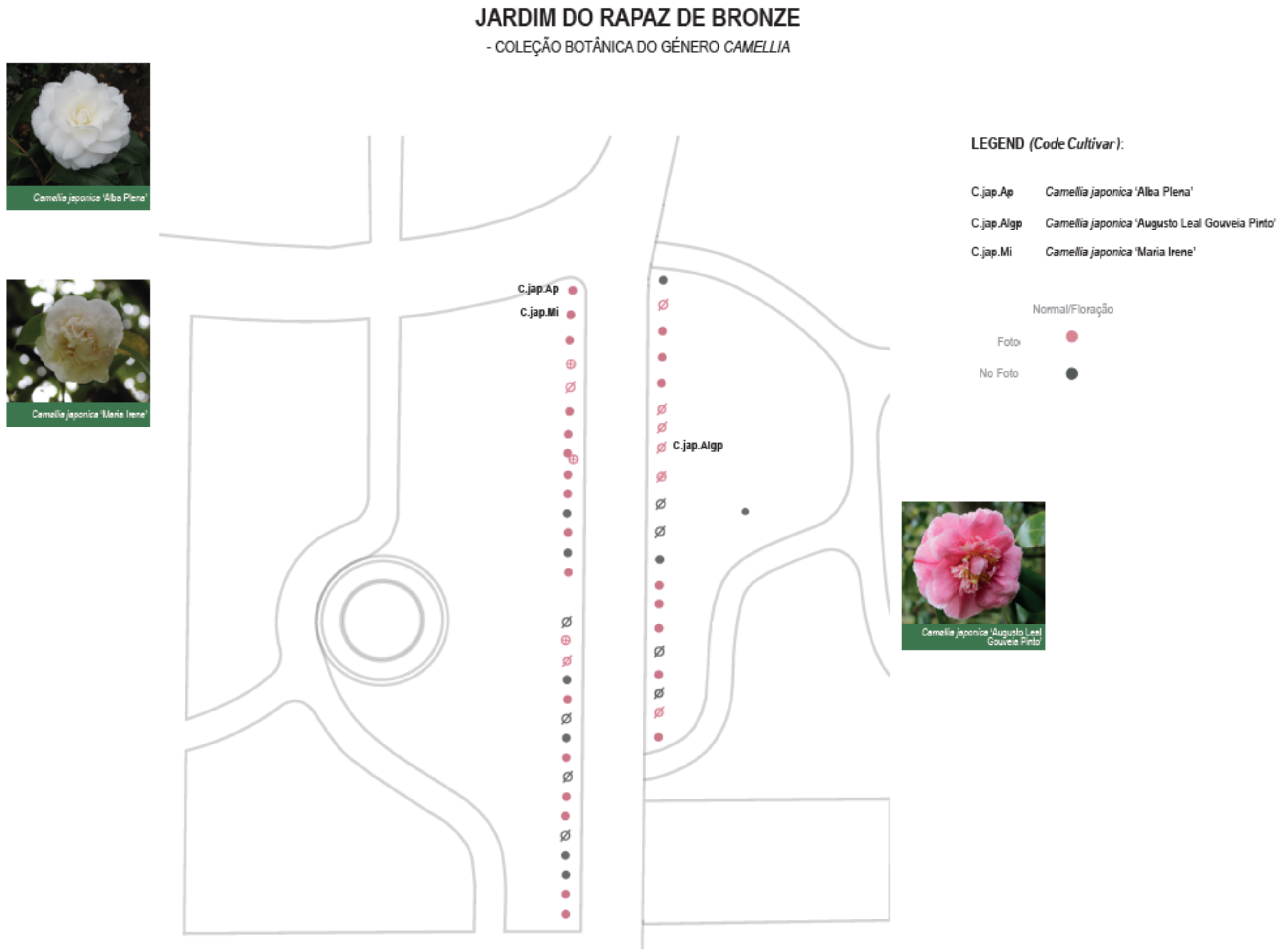

Figure S3. Detailed information about *Jardim do Rapaz de Bronze* and plants localization at Viveiro da Câmara Municipal do Porto (VMP), GPS: 41.155830, -8.558920

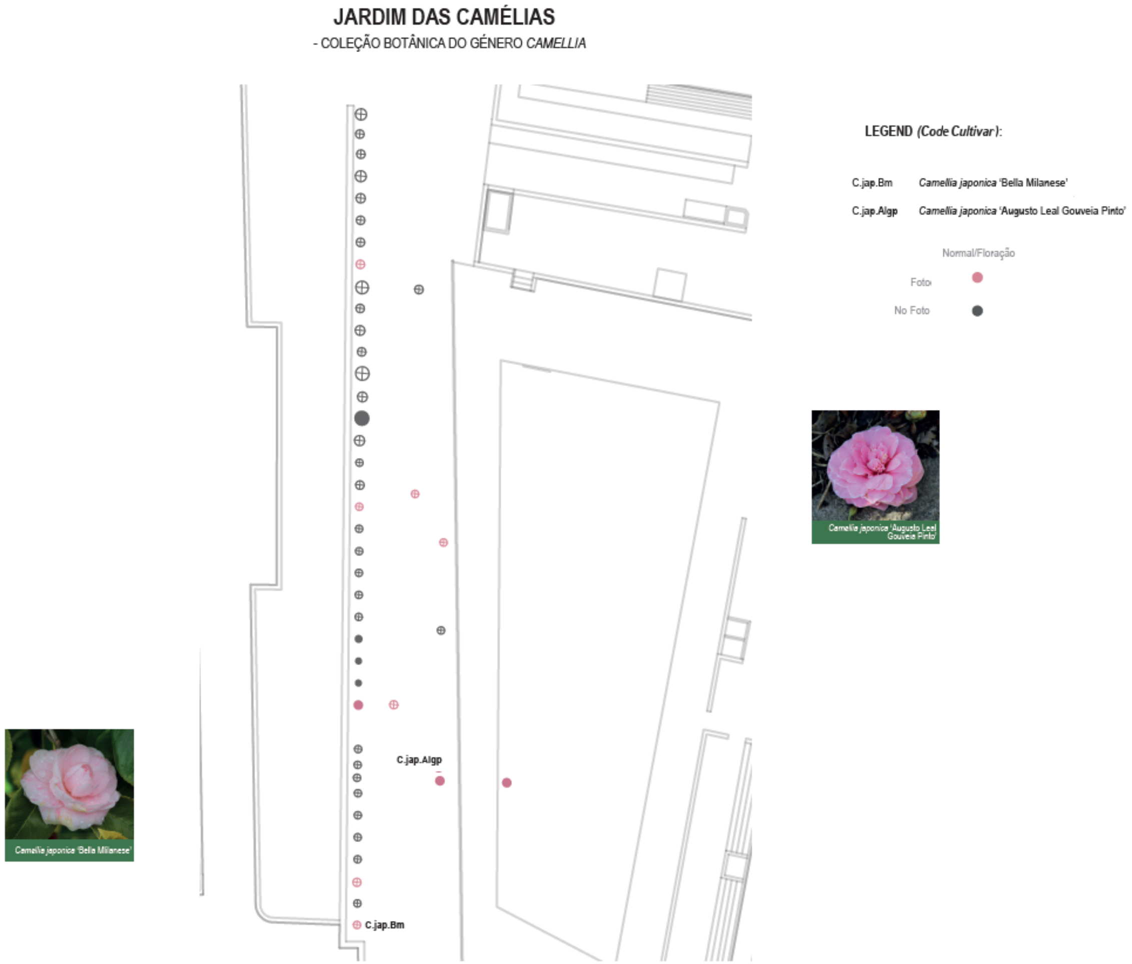

Figure S4. Detailed information about *Jardim das Camélias* and plants localization at Viveiro da Câmara Municipal do Porto (VMP), GPS: 41.155830, -8.558920.

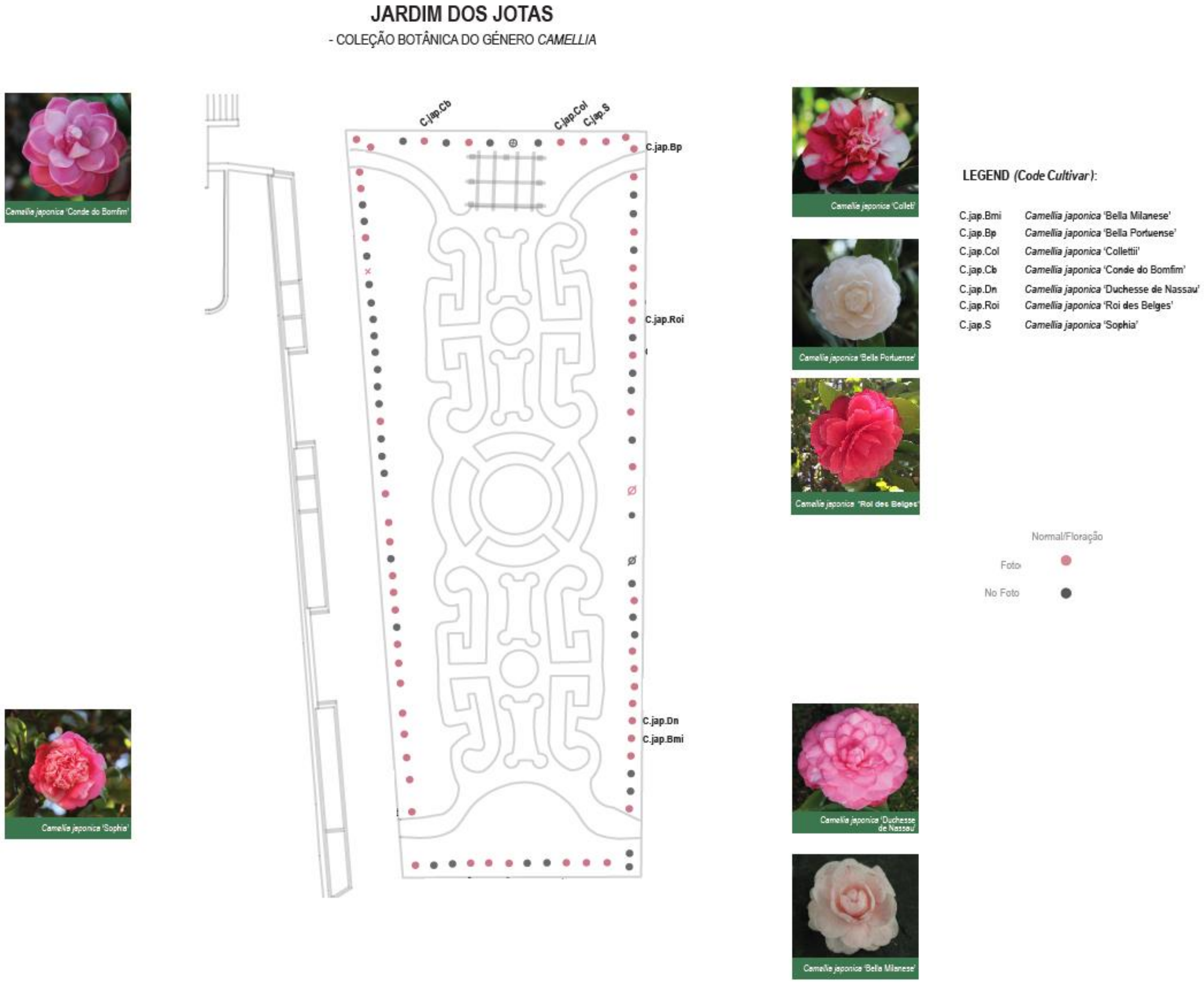

Figure S5. Detailed information about *Jardim dos Jotas* and plants localization at Viveiro da Câmara Municipal do Porto (VMP), GPS: 41.155830, -8.558920

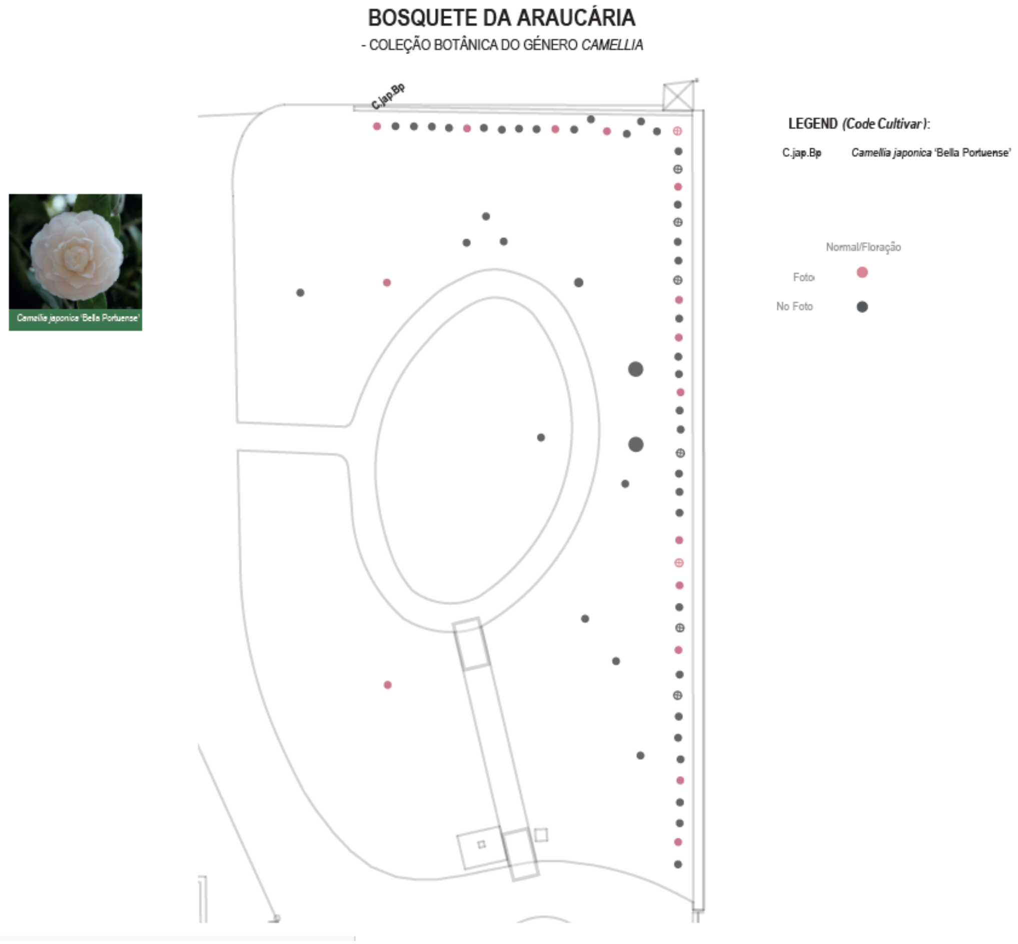

Figure S6. Detailed information about *Bosquete da Araucária* and plants localization at Viveiro da Câmara Municipal do Porto (VMP), GPS: 41.155830, -8.558920.

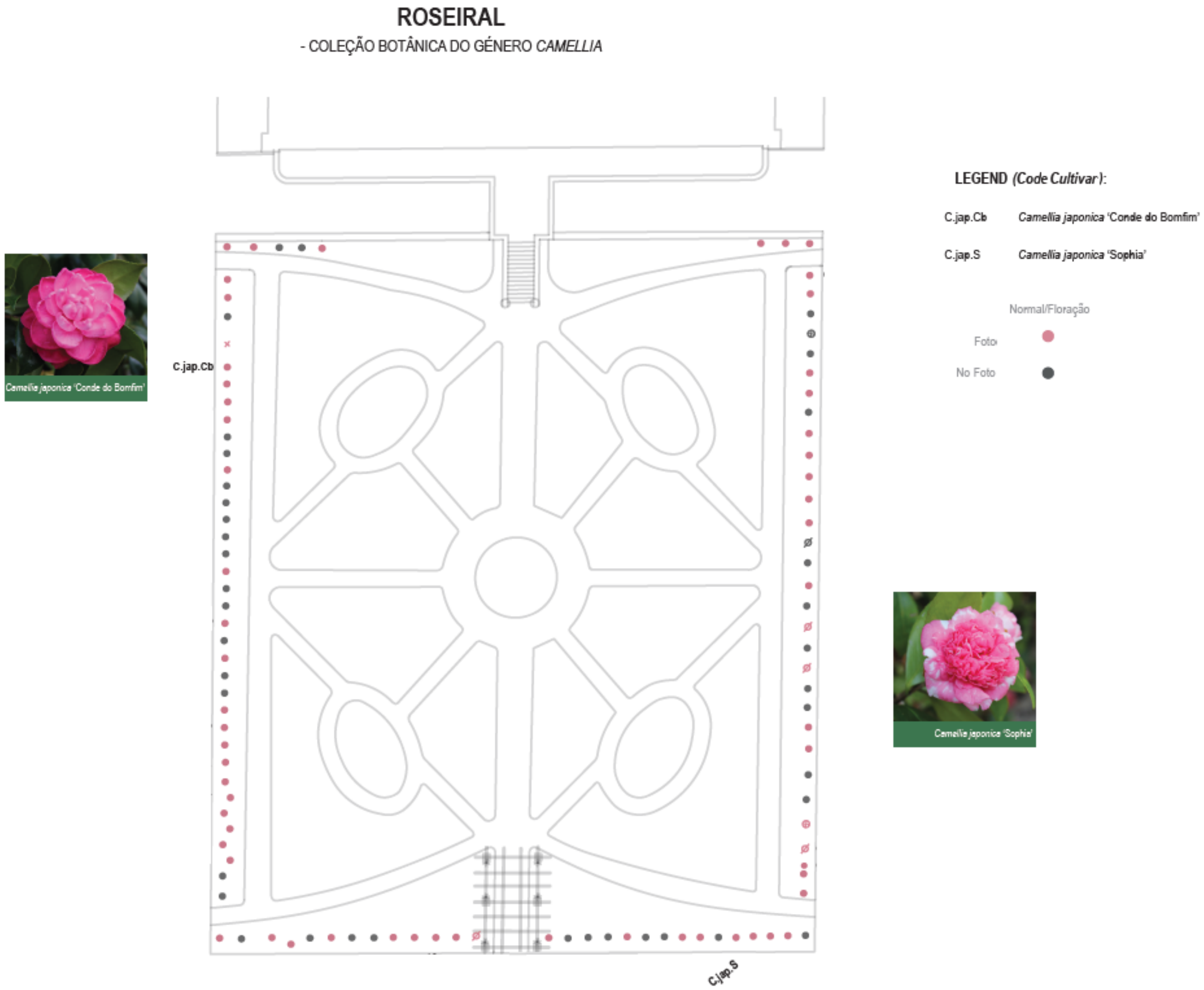

Figure S7. Detailed information about *Roseiral* and plants localization at Viveiro da Câmara Municipal do Porto (VMP), GPS: 41.155830, -8.558920.

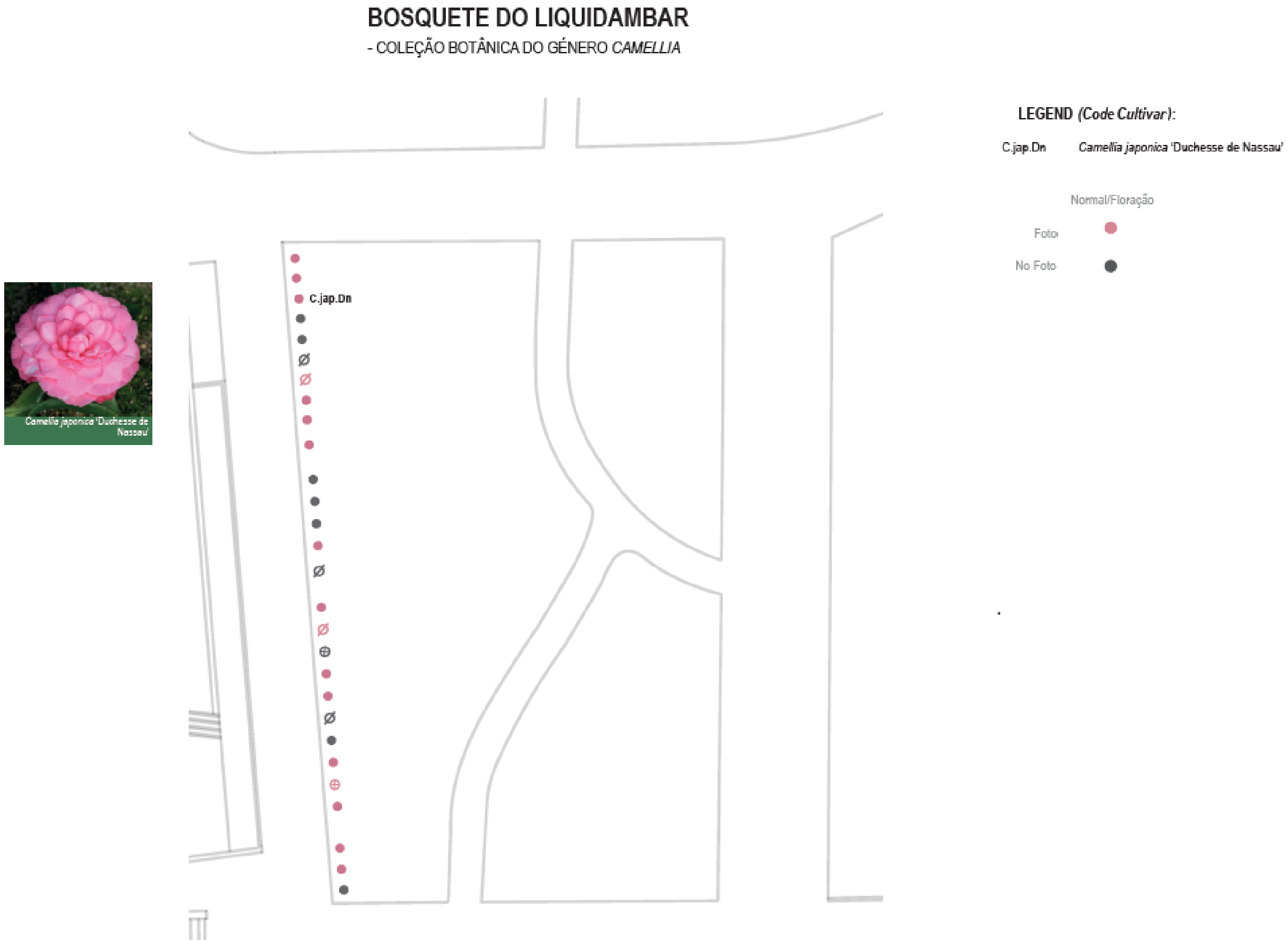

Figure S8. Detailed information about *Bosquete do Liquidambar* and plants localization at Viveiro da Câmara Municipal do Porto (VMP), GPS: 41.155830, -8.558920.

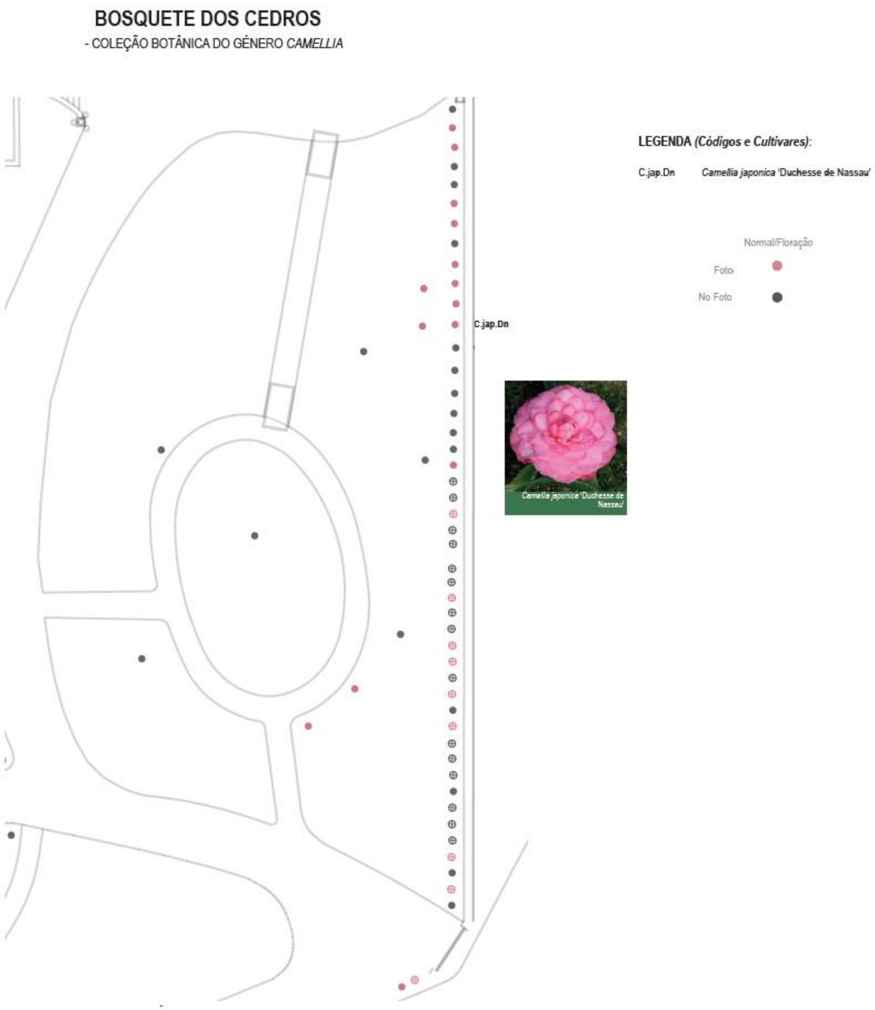

Figure S9. Detailed information about *Bosquete dos Cedros* and plants localization at Viveiro da Câmara Municipal do Porto (VMP), GPS: 41.155830, -8.558920.

Table S2. Detailed information (including first identification and phenotypic description) about *C. japonica* cultivars included in this work.

**Cultivar**	**Detailed information**
**Albino Bottii**	Stefano Pagliai Catalogue, 1867, p.67 as ‘Albeno Botti’: Edged with white and streaked with crimson; imbricated. Illustration Horticole, vol.22, p.587, 1875, pl.ccviii: Clear pink, very large flowers of beautiful form. Fratelli Rovelli, 1896, Catalogue, p.40: Imbricated. A very delicate pale pink with slightly deeper veins, petals edged paler to white. Tirocco, 1928, La Camelia, p.41: Very large flower of a beautiful transparent pale pink petals streaked with carmine. Orthographic errors: ‘Albino’, ‘Albino Bolti’, ‘Albino Battu’, ‘Albina Boti’. Originated in Italy by Botti..
**Alba Plena**	Andrew’s Botanical Repository, 1:pl.25, 1797, as ‘Flore Plena Albo’. Listed again in the 1812 Botanical Repository, vol.10, pl.660; Loddige’s Catalogue, 1814 as ‘Alba Pleno’ and in their 1822 Catalogue as ‘Alba Plena’. The cultivar was brought to England from China in 1792, by Captain John Corner on the East Indiaman, "Carnatic", for the ship’s principal owner, Gilbert Slater. (Farrington, 1999, Catalogue of East India Company Ships’ Journals and Logs 1600-1834, p.105, and Journal and Log of “Carnatic” in the British Library, London). It was taken to USA by Floy in 1800 for John Stevens, New Jersey. The camellia bears a full double, symmetrically imbricated (formal), medium to large size, white flower, showing no stamens and opening flat. It is about 10 cm across and 3.5cm deep. The petals decrease in size towards the centre. A medium to slow grower with light green foliage, leaves ovate. sometimes twisted and turned down at the apex. Sports include: Fimbriata, Mrs Hooper Connell, Mattie R, and Blush Plena. Synonyms: ‘Nankin-shiro’, ‘Da Bai’, ‘Baiyupei’, ‘Baiyang Cha’, ‘Qiayebai’, ‘Napkin Bai’, ‘Baichahua’, ‘Bourbon Camellia’, ‘Double White’, ‘Old White’, ‘Old Double White’, ‘French White’, ‘Thousand Petalled White’, ‘Alba Plena Improved’, ‘Alba Plena Late’, ‘Il Cygno’(Hearn), ‘Alba Pleno’, ‘Alba Plena Ancien’, Alba Plena Imbricata’. See colour photo p.68, Macoboy, 1981, Colour Dictionary of Camellias. Originated in China.
**Augusto Leal Gouveia Pinto**	Real Companhia Horticolo-Agricolo. Portuense Catalogue, No.33, 1899, p.23. No description, and Catalogue No.40, 1906, p.68; Rose form violet pink, petals bordered with white. A white margined form of Grand Sultan, invalidly as ‘Mathotiana’. A very narrow border on each petal. Colour, bright carmine, heavily flushed lavender. The bloom takes on a blue hue in some micro-climates. Jacintho de Mattos, Catalogue No.14, 1900, p.62; Pink bordered with white, rose form. See colour plate in Urquhart, 1956, The Camellia and back cover, RHS., 1988-1989, Rhododendrons with Magnolias and Camellias, No.41. Orthographic variant ‘Augusto Leal Gouvêa’ first mention). Othographic abbreviations: ‘Augusto Pinto’, ‘Augusto Leal’, ‘Augusto Gouveia Pinto’, ‘Augusto L. Gouveia Pinto’, ‘Augusto L’Gouveia Pinto’. For another form of this variety see: Shepherdess. See p.111, black and white photo, Hertrich, 1959, Camellias in the Huntington Gardens, vol.III. Received RHS, Award of Merit, 1958. Received the "William Hertrich Award", 1953 as ‘Jack McCaskill’. Originated in Portugal.
**Bella Milanese**	Luzzatti, 1851, Collezione di Camelia, p.7. No description. Franchetti, 1855, Collezione di Camelie, p.14: Rosy pink, spotted and streaked with pale carmine. Imbricated. Verschaffelt, 1855, Nouvelle Iconographie, Book X, pl.II: Received in the Autumn, 1853 from M. Luzzatti, of Florence, Italy, who obtained it from seed. Blooms, larger than average, are regularly imbricated, having a double form, Those of

	the first rows being rounded, slightly emarginate; those following are lanceolate, at the centre where they are grouped in the form of a rose. Their colouring is a delicate pink, becoming lighter at the margins and variegated with crimson dashes and stripes. Orthographic errors: 'Bella Milanaise', 'Belle Milanaise', 'Belle Milanese', 'Bella Milanesa'.
**Bella Portuense**	José Marquis Loureiro, Catalogue No.1, 1865, p.34, & No.9, 1872-73 p.41: Regular rose form, blush streaked and spotted carmine. Duarte d'Oliveira Jr, 1872, Jornal Horticultura Pratica, 3:230. …medium size rose form, flesh coloured and the first order of petals with a pink hue. Some petals have light blotches or stripes of carmine; however most and especially the outer petals are splashed carmine. Petals are obovat and some of them slightly emarginated at the apex, pertfect imbrications. Leaves are elliptic, acuminate, serrate, medium, uppersurface olive green and lower surface yellowish green. Blooms abundantly. Mercatelli Catalogue 1881, p.11: Large flower; rosy white, of perfect form, petals numerous, transparent, sometimes striped red. Originated in Portugal. Orthographic variant 'Bela Portuense'. Orthographic errors: 'Bella Portuensis', 'Bella Portugesa'. Chinese synonym 'Botusi'.
**Camurça**	Asociasión Española de la Camelia, Camelia, Dec. 2004, p.19. Synonym for Incarnata.
**Colletti**	Jacob Makoy Catalogue, 1838. No description. Jacob Makoy Catalogue, 1841, p.10: "There are many camellias found in commerce which are false (Virus variegated). This is a wonderful variegated, which I have raised from seed." van Houtte Catalogue, 1841, 7:5: Red with white blotches. Most beautiful; then in his 1844-1845, 18:9 Catalogue: Velvety blood red, covered with wide, pure white blotches. Berlèse, 1843, Iconographie, vol.3, pl.275 and Verschaffelt,1850, Nouvelle Iconographie, Book II, pl.I both have illustrations and desciptions: Informal double to peony form, 9 cm across, intense red, marbled white, shows some stamens when fully opened. Large, outer guard petals, imbricated, with small petals in the centre. Mid-season flowering. Rounded, deep green leaves, 7.5 cm long by 5 cm wide, round bases, sharp serrations, slow, bushy growth. Supplied by Jacob Makoy Co., Liège, Belgium who, according to Berlèse, 1843, obtained it from Italy; however Makoy says he raised it from seed in Belgium. Synonyms: 'Collettii Maculata', 'Girard Debaillon', 'Purpliana', 'Purplyana', 'Genevieve de Barbier', 'Tea Garden Strain', 'Collettii Vera', 'Collettii de Milan', 'Collettii Maculosa'. Orthographic errors: 'Colletii', 'Colleti', 'Coletti', 'Colletia', 'Colletti', 'Collestii', 'Colettii', 'Coeltti', 'Coleti', 'Coletti Maculata', 'Colleti Vera', 'Coletti Vera', 'Colleti Maculata', 'Collettia Maculata', 'Collietti', 'Colletti'. Orthographic variant: 'Colettii Vera'. Chinese synonym 'Kelaidi'.
**Conde do Bonfim**	José Marques Loureiro Catalogue No.9, 1872-1873: Formal double, perfectly imbricated. Colour very vivid cherry red. Originated in Portugal.
**Duchesse de Nassau**	Lemaire, 1863, L'Illustration Horticole, pl.376: Light pink with some petals tipped white. Medium large formal double. Mid-season blooming. Originated by Verschaffelt, Ghent, Belgium. Orthographic variants: 'Duchess of Nassau', 'Duchessa di Nassau'. Orthographic errors: 'Duchess le Nassau', 'Duchesse de Nassua'. See coloured pl.346, The Floral Magazine, vol.6, 1867.
**Etoile Polaire**	The Floral World, 1861. No description. Jean Verschaffelt, 1861-1862, Price List, p.35: Imbricated double, full centre, crimson scarlet. Each petal marked at the centre by a white stripe. Obtained from Henderson in sale Oct. 1861. André, 1864, Plantes des Terres

	Bruyéres..., p.243: Very beautifully imbricated flower, carmine pink; all the petals clearly ribboned with metallic white. Illustrated and described in L'Illustration Horticole, vol.13, 1866 under the name 'Stella Polare'. The illustration shows definite bands of rosy white on each petal, while the description reads: "The stripes clearly separate the petals into two equal parts, forming a veritable star." Rollisson's Plant Catalogue, 1877-1878 says: "Crimson, with a white bar through the centre of each petal". Van Houtte Catalogue, 1866-1867, 116:49 is different: Flower a little larger than a C.sasanqua, pale red, incurving, forming a full rose centre and then in his Catalogue, 1877-1878, 174:245: Flower sometimes pink, streaked with red, sometimes light red, imbricated, incurved, rose-form. Then in his 1882-1883, 199:254 Catalogue as 'Stella Polaire', "from Santarelli". Thus it seem that the variety described in SCCS, Camellia Nomeclature is the van Houtte form. The designation "Stella Polare" is Italian for the French "Etoile Polaire" or the English "Pole Star". Orthographic errors include: 'Etoile Polaine', 'Etoile Palaire'. Orthographic variants: 'Stella Polare', 'La Stella Polare'.
**Fimbria Alba**	Transactions of the London Horticultural Society, 1816; Loddige's Botanical Cabinet, 1817. No description. Loddige's Botanical Cabinet, 1826, vol.XII, fol.1103: "There is an uncommon degree of delicacy and beauty in this flower. The original double white camellia is doubtless a most exquisite plant which scarely anything can surpass, yet the one now before us, from the finely fringed edge of the petals, has a novel character peculiarly its own. In the foliage it is scarely, if at all, distinguishable from the double white." (Alba Plena). Originated in China as a sport of Alba Plena. Brought to England in 1816 for Colvill, a nurseryman of King's Road, Chelsea. Illustrations: Mertens & Fontaine, Collection de cent espèces.....camellia, 1845, pl. 46; Macoboy, 1981, The Colour Dictionary of Camellias, p.87; American Camellia Yearbook, 1975 facing p.180; Hertrich, 1954, Camellias in the Huntington Gardens, vol.I, p.135; Front cover, New Zealand Camellia Bulletin, 1975, Vol.IX, No.2. Synonyms: 'Alba Fimbriata', 'Fimbriata Alba', 'Fringed White', 'Alba Plena Fimbriata', 'Double Fringed White', 'Fimbriata Plena', 'Fimbriata Improved', 'Alba Fimbriata', 'Fimbriata Amplissima', 'Fimbriata Alba Plena', 'Fringed Petal'. Orthographic errors: 'Fimbriatta', 'Fimbricata Alba', 'Fimariata'. Although this was an ancient camellia originally imported from China, they now have no record of its original name and have given it the synonym: 'Xiuya Baichilun'.
**Maria Irene**	Alfredo Moreira da Silva, Catalogue No.19, 1964-1965, p.27, ref.108: Very large peony form, creamy white. Haskins Nursery Pty. Ltd. Catalogue, 1964: Creamy white peony form with an anemone centre. Medium large size. Ferreira & Celina, 2000, O Mundo da Camélia, p.96 with colour photo; Flowers mid-season. Originated in Portugal.
**Roi des Belges**	van Houtte Catalogue, 1864-1865, 104:79, as 'Contessa Lavinia Maggi Roi des Belges'. See also van Houtte, 1877, Flore des Serres…,vol.22, p.185, pl.2356-57: A virus variegated form of Lavinia Maggi Rosea; originated by Dominique Vervaene and first exhibited in Brussels about 1864. Its flowers are large, of different colours on the same plant, sometimes self red (Lavinia Maggi Rosea), sometimes red with splashes of white. Synonyms: 'Contessa Lavinia Maggi Roi des Belges', 'Contessa Lavinia Maggi Multicolor', 'Contessa Lavinia Maggi Nuova'. Orthographic error: 'Roi de Belges'.
**Saudade Martins Branco**	Companhia Horticolo-Agricola Portuense, Limitada, Catalogue, No.81, 1942-1943, p.8, ref 1102: Cherry red. Alfredo Moreira da Silva, Catalogue No.78, 1944, p.15, ref 72; Vivid pink, variegated with white. Urquhart, 1960, The Camellia, vol.II, pl.XXXIII: A medium size, semi-double to incomplete double, 10 cm acoss. Bright turkey red, irregularly marbled with white in varying degrees. Petals about 20,

	broad, rounded and entire or shallowly notched, reflexing at the margins and overlapping to form a symmetrical flower. Stamens yellow in a loose central cluster, sometimes intermingled with petaloids. Leaves dark green, oblong-elliptic, 10 cm x 5 cm, apices long tapered, serrations broad and shallow. Blooms mid-season. Colour photos in Catalogues, VII Camellia Exhibition, Porto, 1994, and IV Camellia Exhibition, Santo Tirso, 2001. Originated in Portugal.
**Sophia**	Verschaffelt, 1853, Nouvelle Iconographie, Book IX, pl.II: Very large blooms, with large petals in the first row and a peony form centre, pink with white variegation. Named for the Queen of the Netherlands. Obtained as part of an unknown breeder's collection in Belgium by Glym, Utrecht, Netherlands. Blooms midseason. Habit of growth and colour of flower similar to Elegans, but flower more cup-shaped with lesser petals. Synonyms: 'Sophia No.2, Longview', 'Sophie Glym'.
